# KIXBASE: A comprehensive web resource for identification and exploration of KIX domains

**DOI:** 10.1038/s41598-017-14617-0

**Published:** 2017-11-02

**Authors:** Archana Yadav, Jitendra K. Thakur, Gitanjali Yadav

**Affiliations:** National Institute of Plant Genome Research (NIPGR), Aruna Asaf Ali Marg, New Delhi, 110067 India

## Abstract

The KIX domain has emerged in the last two decades as a critical site of interaction for transcriptional assembly, regulation and gene expression. Discovered in 1994, this conserved, triple helical globular domain has been characterised in various coactivator proteins of yeast, mammals and plants, including the p300/CBP (a histone acetyl transferase), MED15 (a subunit of the mediator complex of RNA polymerase II), and RECQL5 helicases. In this work, we describe the first rigorous meta analysis of KIX domains across all forms of life, leading to the development of KIXBASE, a predictive web server and global repository for detection and analysis of KIX domains. To our knowledge, KIXBASE comprises the largest online collection of KIX sequences, enabling assessments at the level of both sequence and structure, incorporating PSIPRED and MUSTER at the backend for further annotation and quality assessment. In addition, KIXBASE provides useful information about critical aspects of KIX domains such as their intrinsic disorder, hydrophobicity profiles, functional classification and annotation based on domain architectures. KIXBASE represents a significant enrichment of the currently annotated KIX dataset, especially in the plant kingdom, thus highlighting potential targets for biochemical characterization. The KIX webserver and database are both freely available to the scientific community, at http://www.nipgr.res.in/kixbase/home.php.

## Introduction

Transcription, being one of the most fundamental processes in living organisms, is accomplished through stringently regulated assembly of various protein complexes at transcription start sites. Short conserved regions in these proteins often act as independently folding domains that can mediate recognition and transient binding, providing optimal thermodynamic environments and/or docking sites for other proteins leading to the successful formation of the transcriptional machinery. The kinase-inducible domain interacting (KIX) domain, approximately 90-residues in length, is one such example reported to play an indispensable role in transcription^1^, by binding to a variety of transcription factors, leading to stabilisation of protein-protein interactions during assembly of the transcription apparatus^2–5^. Structurally, KIX domains are triple helix bundles, with hydrophobic allosteric sites for various transactivation domains (TADs) of transcription factors like CREB-pKID^3^, Mixed lineage leukaemia protein (MLL)^6^, FOXO3a^7^, breast cancer 1 (BRCA1)^8^, c-Myb^9^ and sterol responsive element binding proteins (SREBP)^10^. The KIX domains not only serve as docking sites for TADs but also assist in proper folding of intrinsically disordered TADs. Studies have shown direct involvement of KIX domains in many biological processes like fatty acid metabolism^11^, multi-drug resistance^2^, and long-term memory storage in mammals^12^. KIX domains provide a paradigm for research on molecular allostery, as well as coupled folding and binding mechanisms with TADs. Interaction of KIX with various proteins is important for normal cell function and thus overall health^12^. For example, KIX binding with MLL protein is an important step in MLL mediated gene expression and the consequent production of normal blood cells. KIX is the binding partner of MLL-activation domain, that is lost due to chromosomal translocations causing childhood and therapy-induced leukemia^13^. The importance of KIX domains has been well established and is an active research area not only in mammals but also in yeast and plants^2,11,14^. Although KIX domains are yet to be experimentally characterised in the plant kingdom, the global protein databases like InterPro^15^ reveal their presence in large numbers in many plant species, including *Arabidopsis thaliana*, *Oryza sativa*, *Medicago truncatula*, *Populus trichocarpa, Glycine max*, etc. Recent taxon-specific computational studies have also suggested the presence of KIX domains in Arabidopsis and rice homologs of MED15 and CBP related Histone acetyl transferase (HAT) proteins as well as F-box proteins of rice^14,16,17^. Functional inferences have been reported in plants where mutations in KIX expressing genes led to multiple development and phenotypic anomalies^18^. Expression studies followed by SNP analysis revealed the association of KIX domain with stress response, leaf maturation, floral development and seed development^19,20^.

Despite the characterization of three major classes of KIX, (based on association with CBP, MED15, or RECQL5 helicases), there is no web resource that caters to these domains, their identification or comparative analyses. Furthermore, the major global protein domain databases like InterPro^15^, Pfam^21^ or Prosite^22^, when queried for KIX, return predominantly the first of KIX sub-classes, i.e. KIX domains found in CBP related proteins, and very few MED15 associated KIX domains. Most notably, there is a complete omission of RECQL5 associated KIX family members in the general sequence databases. RECQL5 KIX homologs have been reportedly identified in plants as well^14^, but no records can be found for this in any public database. For example, human MED15 and human RecQL5 proteins, both of which contain an experimentally characterized KIX domain along with availability of 3D structure (PDBIDs ‘2GUT’ for MED15 and ‘4BK0’ for RECQL5), do not show presence of any KIX domain when queried by InterPro or Pfam. Similarly, in plants, InterPro shows the presence of KIX domains in very few MED15 family members of *Arabidopsis thaliana* whereas KIX domains have been identified not just in members of Arabidopsis Histone acetyltransferase (HAC) family [namely HAC1 (AT1G79000), HAC5 (AT3G12980), HAC12 (AT1G16710)], but also in CBP/P300 like protein (At1g16705), HAC related protein (At4g32295), RECQL3 protein (AT4G35740) and an uncharacterised protein (At3g24150)^14,16^. One possible reason for this omission may be that KIX domains tend to have higher sequence similarity within their own class. Sequence homology is not only difficult to detect between KIX sub-classes but also between taxonomic kingdoms of the same sub-class i.e. between MED15 associated KIX of fungi, metazoans and plants^1,14^. Therefore, the conventional methods of local sequence similarity search tend to fail when trying to identify KIX domains among diverse organisms across different kingdoms of life. This lacuna in accurate KIX identification inspired us to design an interactive web platform, KIXBASE, which would provide a platform for detecting the presence of KIX domains as well as a comprehensive online repository of KIX in metazoans, fungi and plants developed through detailed computational meta-analysis of available data in resources like the NCBI RefSeq^23^, UniProt^24^ and Phytozome^25^, followed by HMM based domain identification and prediction along with assessment of features like structural propensities, class-based residue conservation and domain organization. To our knowledge, KIXBASE is the only server that can identify all three classes of KIX domains, including those in CBP, RECQL5 and MED15 containing proteins, whereas no other program can presently do so. The web interface of KIXBASE is user-friendly, providing links to explore multiple features of each KIX entry. All the sequence records are in standard FASTA format and have been further validated through other resources viz. MUSTER^26^, PsiPred^27^ and Clustal Omega^28^. We describe the various capabilities and features of KIXBASE, along with a specific case study. In summary, this work massively expands the known KIX domain repertoire and provides evidence for structural conservation across all three classes of KIX domains despite low conservation in sequence across various life forms. We show that KIX domains maintain conservation of secondary and tertiary structure, as well as very similar spatial locations of intrinsically disordered regions. The database is continually updated and maintained in the laboratory. The KIX domain database has been developed using a combination of public resources (MUSTER, PSIPRED, IUPRED, HMMER) and in-house shell scripts, perl functions, python and R code. KIXBASE presently contains 2526 records of KIX domains representing 581 organisms from metazoans, fungi and plants.

## Results

### KIX domain identification

KIXBASE represents a compilation of KIX domains predicted by a search algorithm based on hidden markov model profiles trained on known KIX domains as described in Methods. The input sequences used for training the profile HMMs can be retrieved from the KIXBASE website. The search algorithm was tested for predictive accuracy in identification of KIX domains in higher organisms, as described in the Benchmarking section below, and was found to be highly accurate. Based on the superiority of our search algorithm as compared to other existing programs, it was developed into a predictive online web server, which was used to scan the full proteomes of all available metazoans, plants and fungi (581 organisms in all). The resulting 2526 KIX domain records along with the web server were assembled into a single browseable online platform, namely KIXBASE. The following sections describe this database and associated features that enable a through investigation of the largest online KIX domain repertoire available to date for higher organisms. We thus present KIXBASE as the only dedicated database, as well as analysis portal for KIX domains, wherein, apart from identification of new KIX, additional features like secondary structure and disorder prediction, extraction of KIX regions, alignments and scoring etc. are assembled on a single platform.

### KIXBASE design and content

**Figure 1:**
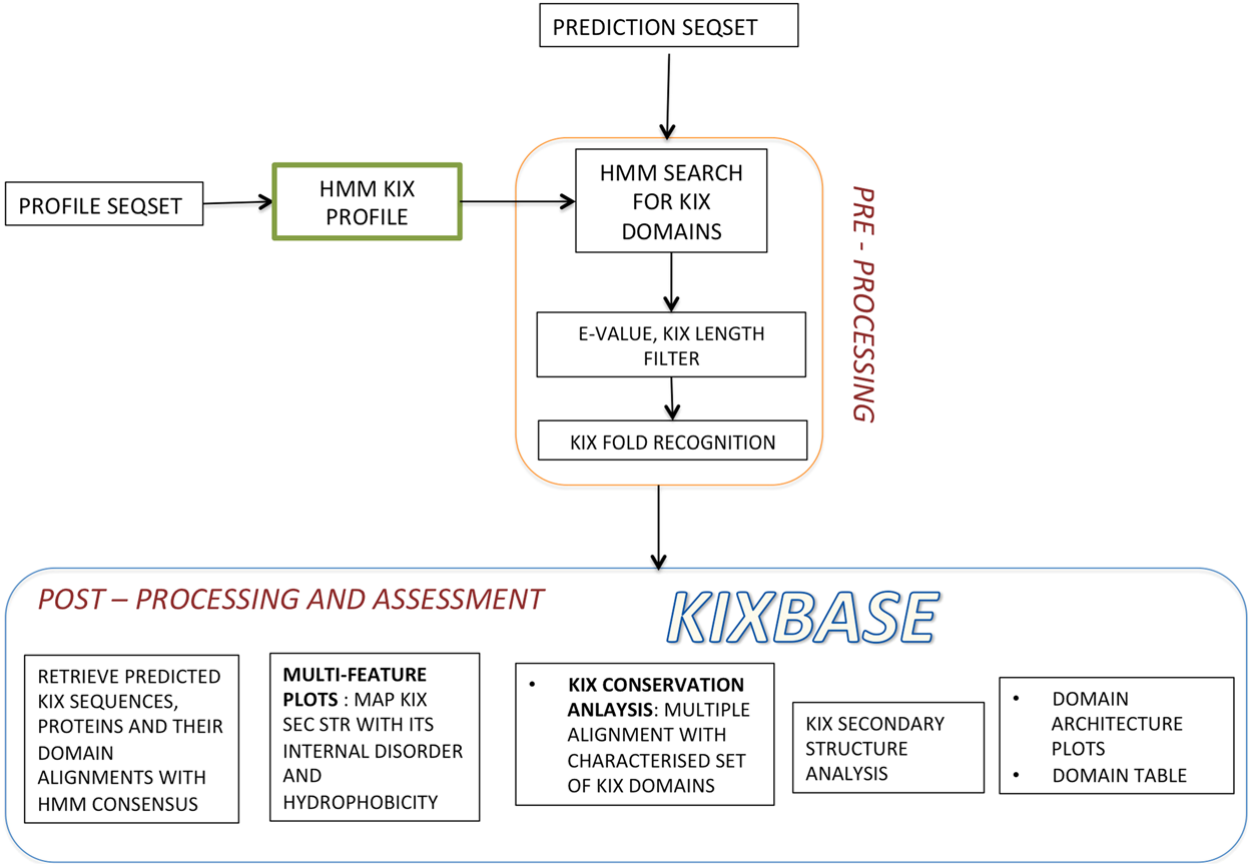
KIXBASE Workflow. Flowchart depicts KIX prediction algorithm adopted by KIXBASE which is made stringent for false positives in the pre-processing step by use of filters at primary sequence level like the E-value cutoff of 0.05, KIX length >=70 and structure level through tertiary fold recognition match with any of the PDP KIX domain. After the prediction of KIX domains, post processing includes analysis of all KIX domains at various conservation, structural and functional level.

**Figure 2:**
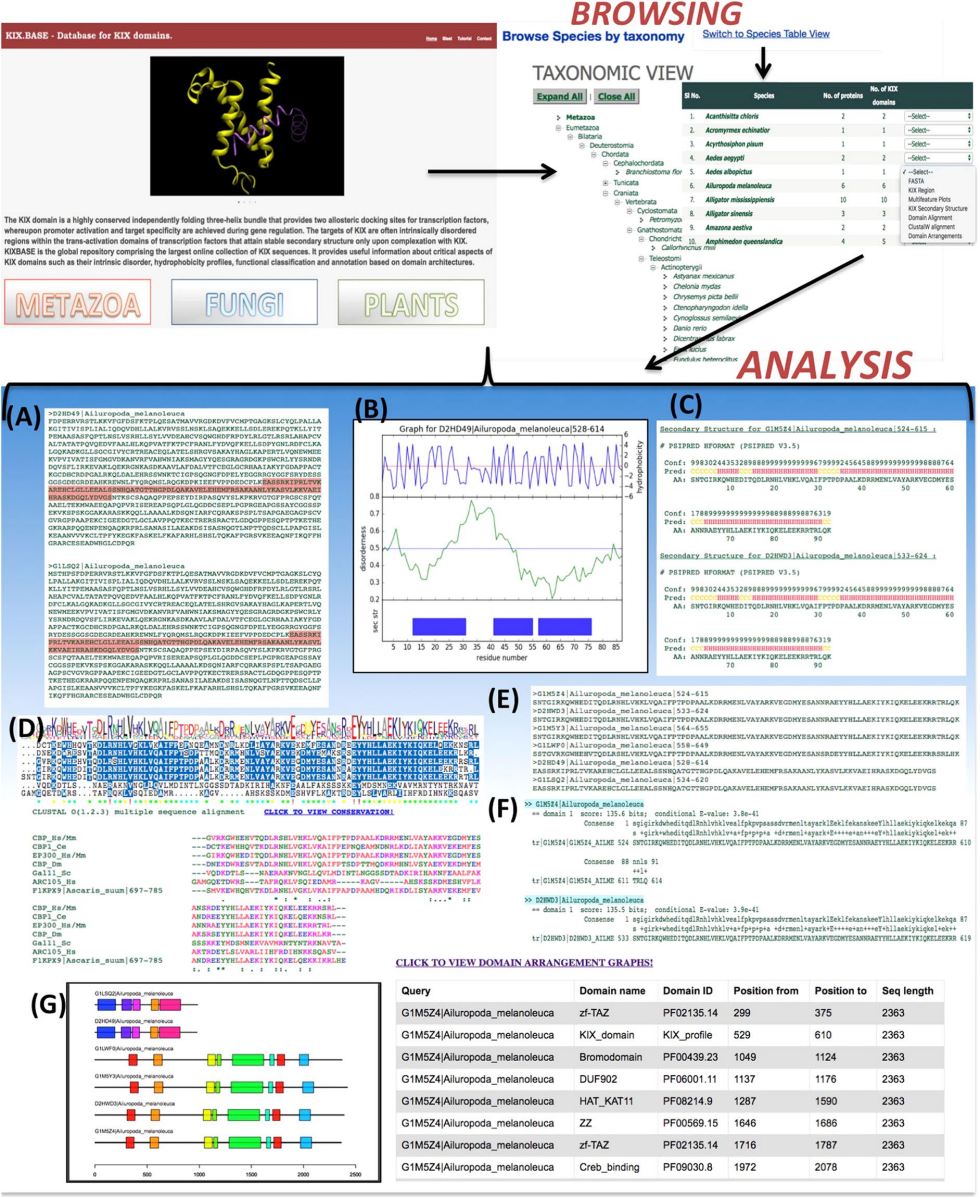
KIXBASE Screenshots. (**A**) Fasta with highlighted KIX domains, (**B**) Multi-feature plots depicting secondary structure of KIX mapped with internal disorder and hydrophobicity, (**C**) Secondary structure prediction of KIX, (**D**) Conservation of predicted KIX with characterised KIX domains, (**E**) Extracted KIX domains, (**F**) Domain alignment with HMM consensus, (**G**) Domains present in KIX proteins in domain organisation plots and tabular format.

Figure 1 shows the overall design and workflow of KIXBASE, while the actual web page along with its data browsing and analytical capabilities are depicted as screenshots in Fig. 2. As can be seen in this figure, the homepage provides direct navigation to KIX domains in the major taxonomic kingdoms, namely metazoans, fungi and plants. Within each of these three main sections, users can browse KIX records based on the taxon based classification tree or by name of species of interest. Information for species based records can be retrieved, extracted and viewed in several different ways, including KIX region (highlighted on the full protein sequence), FASTA format, Multi-feature Plots (Secondary structure, intrinsic disorder and hydrophobicity), structural propensity (secondary element prediction), extent of conservation, and domain architectural arrangements, as shown in Fig. 2A–G. In addition to browsing, users can also test their own sequence(s) of interest, if any, for potential KIX domains through the HMM based predictive web server that was used to scan all proteomes. If a KIX region is found, it will be highlighted on the submitted sequence. Users can also quick search for KIX domains across the entire database through BLAST link provided in the navigation bar. The resulting hits, if any, will be returned in a tabular format, with the option to sort and download the data or retrieve individual alignments. ‘FASTA’ is the simplest view highlighting the predicted KIX domains on the FASTA sequences of the corresponding proteins (Fig. 2A). The ‘multi-feature plots’ output contains informative plots, which map secondary structure of KIX region with its intrinsic disorder and hydrophobicity (Fig. 2B). It has been reported in certain studies that 3^rd^ helix of KIX domain is partially disordered in unbound form and gains helicity upon binding with TAD and formation of ternary complex, signifying a direct relation of helical propensity with cooperative binding^29,30^. These plots provide enhanced visualisation of KIX region in terms of its structural properties. The ‘Get secondary Structure’ option on the navigation bar contains the secondary structure output of every predicted KIX domain. This option provides important information for annotation, as the KIX domain exists as a triple helix interrupted by loops or 3–10 helices. The secondary structure output contains three parameters i.e. Conf (Confidence), Pred (Predicted secondary structure) and AA (Target Sequence), as shown in Fig. 2C. The Conf line contains a series of numbers, which represent the confidence of a corresponding secondary structure prediction ranging from low to high (0 = lowest, 9 = highest). The Pred line contains three characters i.e. H, E and C where H represents helix, E as sheet and C as a coil for the particular amino acid. The AA line is the target amino acid KIX sequence. A single protein may contain one or more KIX domains, therefore, the KIX boundaries (start-end) are added in the identifier line of each predicted KIX domain. The ‘Clustal Omega alignment’ displays multiple sequence alignments for every KIX domain with a core set of experimentally characterised KIX domains from different proteins in two distinct modes namely Clustal-O color format, and sequence conservation based display (Fig. 2D). This further helps in the annotation of predicted KIX domains through insights into the identity, similarity and conserved regions of new KIX sequences with characterised domains For e.g., asterisk (‘*’) symbol represents complete identity at a position whereas colon (‘:’) and period (‘.’) represents conservation between strongly and weakly similar amino acids respectively in an alignment as per PAM250 scoring matrix. The ‘KIX region’ link directs the user to the page containing KIX domains predicted from the input FASTA sequences (Fig. 2E). The ‘domain alignment’ option displays the HMM alignment of the predicted KIX domain with consensus sequence derived from the profile HMM. The score and *E-value* gives an idea about the probability of the KIX prediction to be a significant hit (Fig. 2F). The ‘Domain arrangements’ option is the most important analytical feature for KIX domains as it gives information about the other conserved domains present along with KIX domain in a particular entry (Fig. 2G). KIXBASE thus helps in identification, domain architecture and functional analysis of uncharacterised KIX proteins especially in the plant kingdom, where it is least characterised.

### Benchmarking of KIXBASE

As described in Methods, the Jackknife test was used to evaluate the performance of KIXBASE, as this test is considered to be the least arbitrary and most objective as compared to other cross-validation methods that evaluate quality of computational models, namely sub-sampling (or n-fold cross-validation) test, and the independent datasets^31^. For this experiment, two sets of complementary sequences were used, namely training data set and testing dataset. Performance was tested on both sets, including positive sequences that were not used for generating the profile (pseudo-novel sequences), as well as a negative dataset that included KIX structural homologs in order to incorporate an additional level of stringency towards accurate identification. The Jackknife test revealed very good statistical parameters, namely a perfect sensitivity of 100%, accuracy >96%, and a Matthew’s Correlation Coefficient (MCC) of 0.94. Encouraged by such a superior performance, we carried out an objective sequence-by-sequence assessment of performance of KIXBASE with other existing methods available publicly.

**Figure 3:**
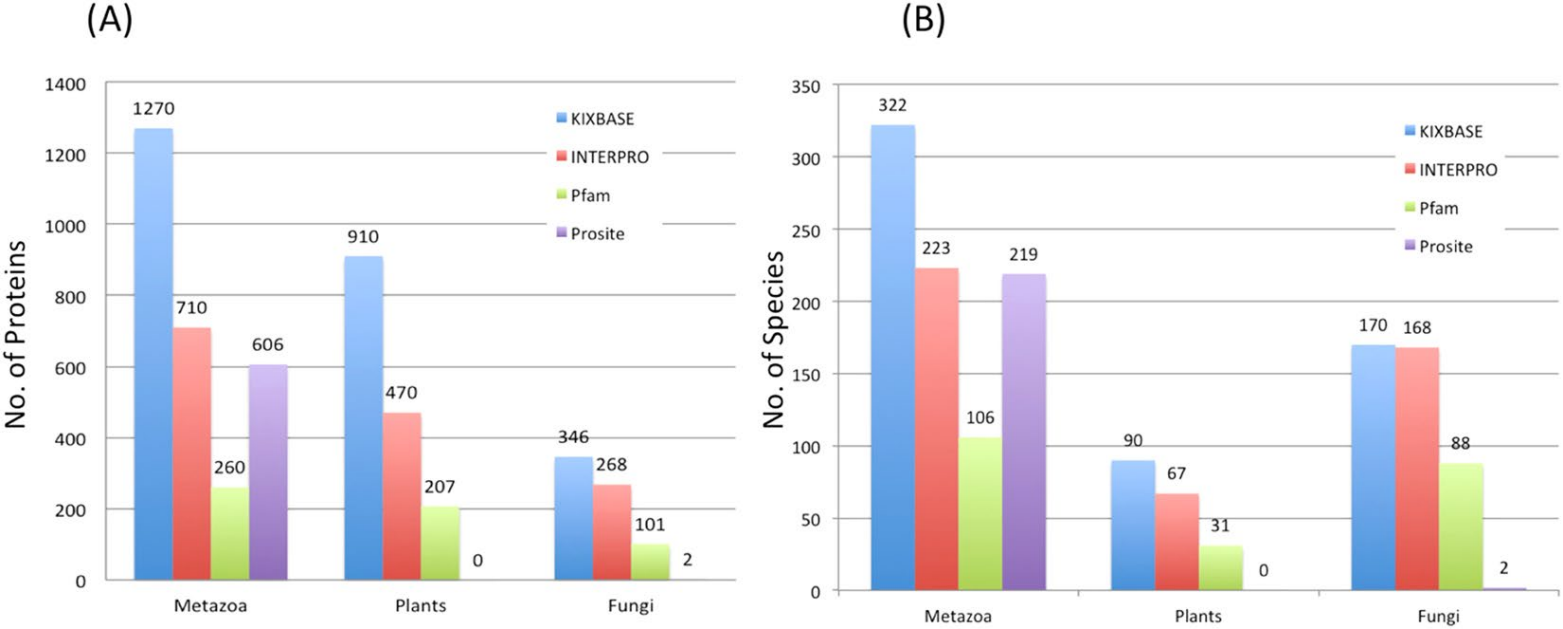
KIXBASE Benchmarking. KIX records from KIXBASE were compared with the recent release of InterPro, Pfam and Prosite. KIXBASE currently have the highest number of KIX entries both in terms of number of proteins and number of species as compared to other three databases, owing to its power to identify more variable and unique domains.

Figure 3 depicts such a comparative evaluation with three major global protein databases, namely Prosite, Pfam and InterPro. As can be seen in the Fig. 3A, KIXBASE has significantly more data records for KIX domains than any other resource. In addition, the Fig. 3B shows KIXBASE to contain a much wider representation of species than any of the other databases. The number of entries for KIX predictions, both in terms of number of species and number of proteins, in every taxonomic group, is much higher as compared to other databases, and we checked the veracity of these predictions by comparing the results for known KIX domains. As mentioned earlier in the Introduction section, the human MED15 and human RecQL5 proteins (both of which contain an experimentally characterized and structurally resolved KIX domain) do not show presence of KIX when queried by InterPro or Pfam. However, KIXBASE reveals the presence and position of KIX in both these sequences. Similarly, in plants, InterPro shows the presence of KIX domains in very few MED15 family members of *Arabidopsis thaliana* whereas KIX domains have been identified and characterised in several other proteins, all of which are identified and represented in KIXBASE. We ascribe this superiority of domain identification of our platform to the rigorous profile HMMs that have been used for training in KIXBASE.

**Figure 4:**
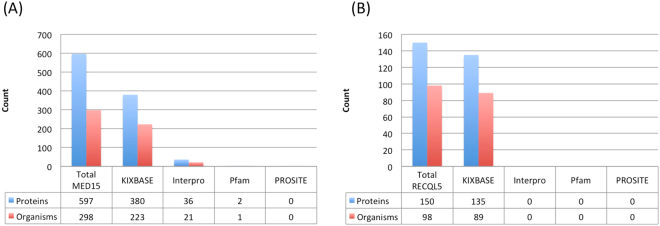
KIX Domain Prediction for MED15 and RECQL5 containing proteins of metazoans. Here we have compared the number of KIX domain containing proteins that can be correctly assigned by four programs, namely KIXBASE, InterPro, Pfam and Prosite, from among the known MED15 (panel A) and RECQL5 (panel B) containing proteins. The lower panel shows the taxonomic classes of these proteins.

Interestingly, plants have the highest density of KIX domains per genome, as compared to fungi or metazoans. Most notably, as mentioned before, KIXBASE is the only web resource providing annotation for RECQL5 helicase associated KIX domains. This can be seen better in Fig. 4, panels A and B, where we performed a comparative analysis for prediction of KIX domains in MED15 and RECQL5 sequences of metazoans through KIXBASE, Pfam, InterPro and prosite, in order to further benchmark the program. As can be seen from both panels of Fig. 4, it is clear that for MED15 and RECQL5 categories of metazoans, KIXBASE is the only platform that can identify KIX domains, while the existing global repositories do not identify these domains at all. For example, of the 597 proteins known to contain a MED15 domain (Interpro ID IPR019087), KIX was identified in 380 by KIXBASE, as compared to merely 36, 2 and zero by the other databases (Fig. 4, panel A). The case of RECQL5 is even worse, where the general databases completely fail to identify KIX domain within known RECQL5 containing proteins (Interpro ID IPR010716), in sharp contrast to KIXBASE, irrespective of any class of organisms, as shown in the lower panel.

However, no comparative performance evaluation is complete without a statistical test using a common benchmark protocol. A pre-requisite for such a test would be the original sequences that were used to build the respective profiles, followed by regeneration of the hidden markov model. Pfam, having two distinct KIX domain profiles meets this requirement, and therefore, we were able to perform a Jackknife test on Pfam as described in Methods for KIXBASE benchmarking.

However, InterproScan and Prosite, being consortia of independent domain databases like the CDD, do not have their own KIX profiles. Pfam, on the other hand, has two profiles for KIX, [namely Pfam-KIX and Pfam-KIX-2]. We downloaded the full FASTA formats from multiple sequence alignment (MSA) of both these Pfam entries, and performed a Jackknife re-sampling of the aligned sequences, using *hmmbuild* program from the HMMER suite.

Table 1 shows the outcome of this test, and KIXBASE can be seen to have a higher sensitivity, accuracy and MCC than Pfam. However specificity and precision of both Pfam profiles is better, implying that KIXBASE is stronger at identification of true positives, while Pfam succeeds in evading false positive hits. This aspect may explain why Pfam failed to detect multiple classes of KIX as well as some of the experimentally characterized KIX domains, as was observed in Figs 3 and 4. The high precision of Pfam also reflects a bias towards the initial seed set of KIX that were used to make the model. Pfam profiles are class-specific, made from greatly stringent datasets. Unlike KIXBASE, which was built on 312 sequences representing all three classes of KIX, the two Pfam profiles were generated using limited datasets; The first KIX profile, showing 100% precision on the Jackknife, has exactly five sequences in the dataset, all of which are CBP-KIX specific. The second profile is MED15-KIX specific and has 77 sequences. In view of this, we made a further attempt to compare performance across these three profiles generated using variable sub-classes.

**Table 1:** Comparative Performance of KIXBASE and Pfam HMM profiles using Jackknife test.

	KIXBASE	Pfam_KIX	Pfam_KIX_2
Specificity	96.85	100	98.86
Sensitivity	100	82.73	90.46
Accuracy	97.65	95.62	96.73
Precision	91.50	100	96.42
MCC	0.94	0.88	0.91

For a true comparative evaluation, we selected test sequences whose fraction of KIX proteins (positive set) could be treated as similar to the actual frequency of KIX proteins in nature. Accordingly, we tested KIXBASE and both Pfam profiles on subsets of yeast, Arabidopsis and human proteomes, where known KIX-containing proteins were treated as the positive set sequences, while the negative set sequences were proteins known ‘not’ to contain any KIX. Thus, our positive test set contained known Med15, CBP and RECQL proteins having complete/partial KIX domain from uniprotKB. The negative test set contained all reviewed (swissprot) protein sequences with molecular function GO term ‘not’ related to transcription. Results of the stastistical test are shown in Table 2.

**Table 2:** Comparative Performance of KIXBASE and Pfam HMM profiles using independent dataset.

	Human			*Arabidopsis thaliana*			*Saccharaomyces cereviseae*		
	KIXBASE	Pfam KIX	Pfam KIX_2	KIXBASE	Pfam KIX	Pfam KIX_2	KIXBASE	Pfam KIX	Pfam KIX_2
Sensitivity	84.21	36.84	26.32	75	3.57	32.14	100	100	100
Specificity	100	100	100	100	100	100	100	100	100
Precision	100	100	100	100	100	100	100	100	100
Accuracy	99.98	99.93	99.91	99.94	99.79	99.85	100	100	100
MCC	0.64	0.42	0.40	0.61	0.13	0.40	0.7	0.7	0.7

As can be seen from this table, KIXBASE performs much better than (or equal to) Pfam in all measures evaluated. As mentioned previously, the two Pfam KIX profiles are very sub-class specific, one for CBP-KIX domains and the other for MED15-KIX of yeast and plants. Consequently, these profiles are unable to identify any KIX domain outside of these classes or taxa. The perfect score of Pfam in case of yeast merely reflects the absence of any experimentally characterized RECQL-5 domains in this organism to date, and consequently RECQL-5 identification was not used for performance evaluation in yeast, providing an example of how difficult it is to reveal the true nature of Pfam deficiency in case of KIX. However, all three classes of KIX are experimentally characterized in humans with structures available in PDB, thereby making it possible to further elaborate the class-wise performance of all three profiles for humans, as shown in Table 3.

**Table 3:** Number of experimentally characterised human KIX predicted by KIXBASE and Pfam out of total proteins in uniprotKB.

	KIXBASE	Pfam_KIX	Pfam_KIX_2
CBP KIX	7/8	7/8	5/8
Med15 KIX	7/9	0/9	0/9
RECQL5 KIX	2/2	0/9	0/9

As can be seen from this table, only KIXBASE can predict all classes of KIX domains whereas both Pfam KIX profiles are only able to identify CBP-KIX. It may be noted here that although Pfam_KIX_2 profile is based on MED15-KIX specific sequences (of yeast and plants), it is still not able to classify Med15 KIX in humans (highlighted in Red font in Table 3).

### KIXBASE: A Case Study

Since the KIX domain is an independently folding domain serving as a docking site for several transcription factors, we analysed KID/c-Myb binding site in mouse CBP KIX through the multi-feature plot option available in KIXBASE. This feature maps hydrophobicity of KIX residues with their internal disorder and secondary structure prediction making it convenient to observe and analyse these three important structural properties with respect to each other. In KIX domain, KID and c-Myb bind to a hydrophobic pocket defined by specific alpha-1 and alpha-3 helix residues^32^.

**Figure 5:**
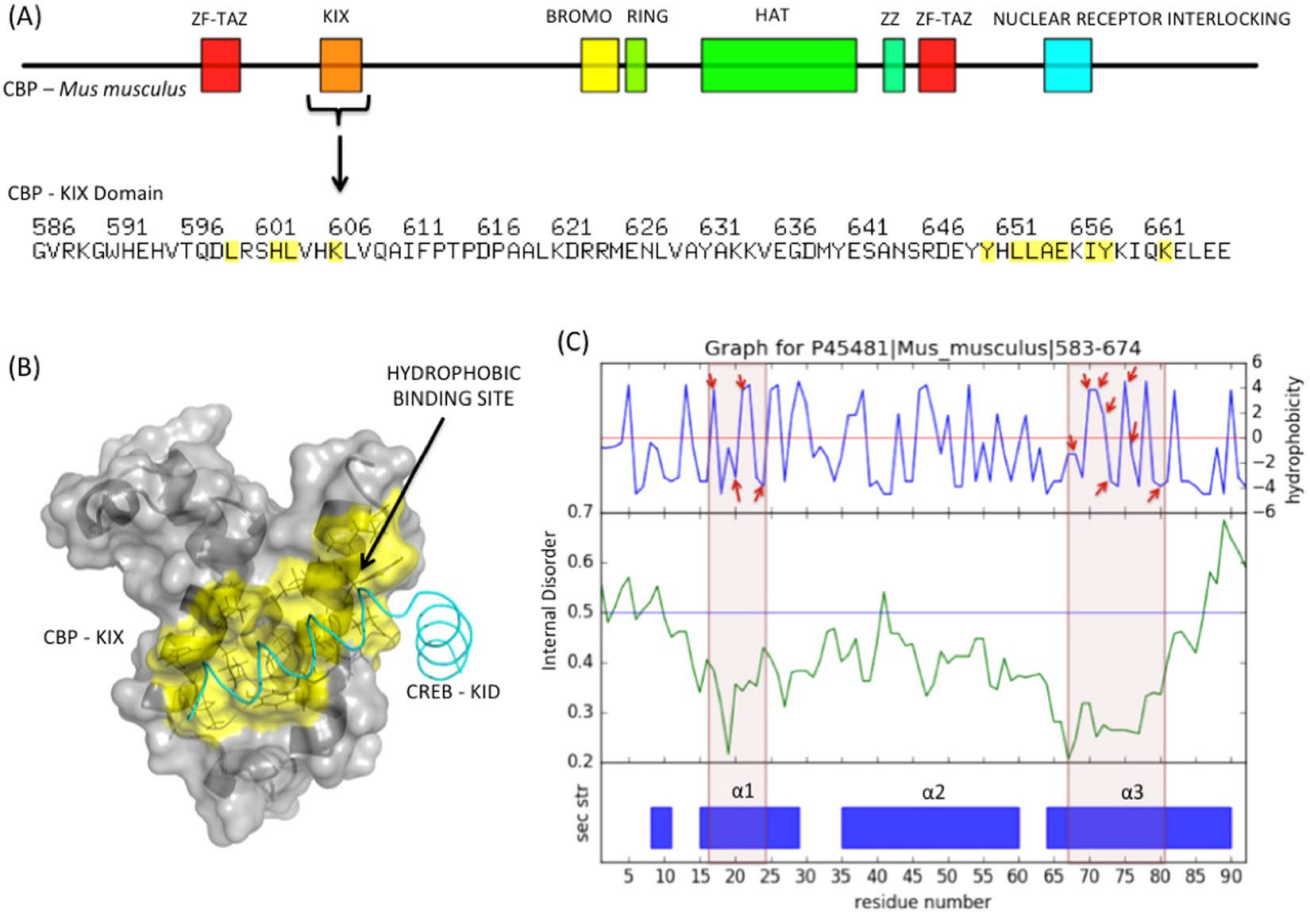
CREB-KID binding site analysis in CBP-KIX domain through multi-feature plot output in KIXBASE. (**A**) Domain arrangements in mouse CBP protein. KIX sequence highlighting residues that bind to CREB-KID. (**B**) Solution structure of CBP-KIX bound to CREB-KID, yellow region represents hydrophobic binding site. (**C**) KID binding residues marked as red arrows in hydrophobicity plots, mapped to their internal disorder and secondary structure.

**Figure 6:**
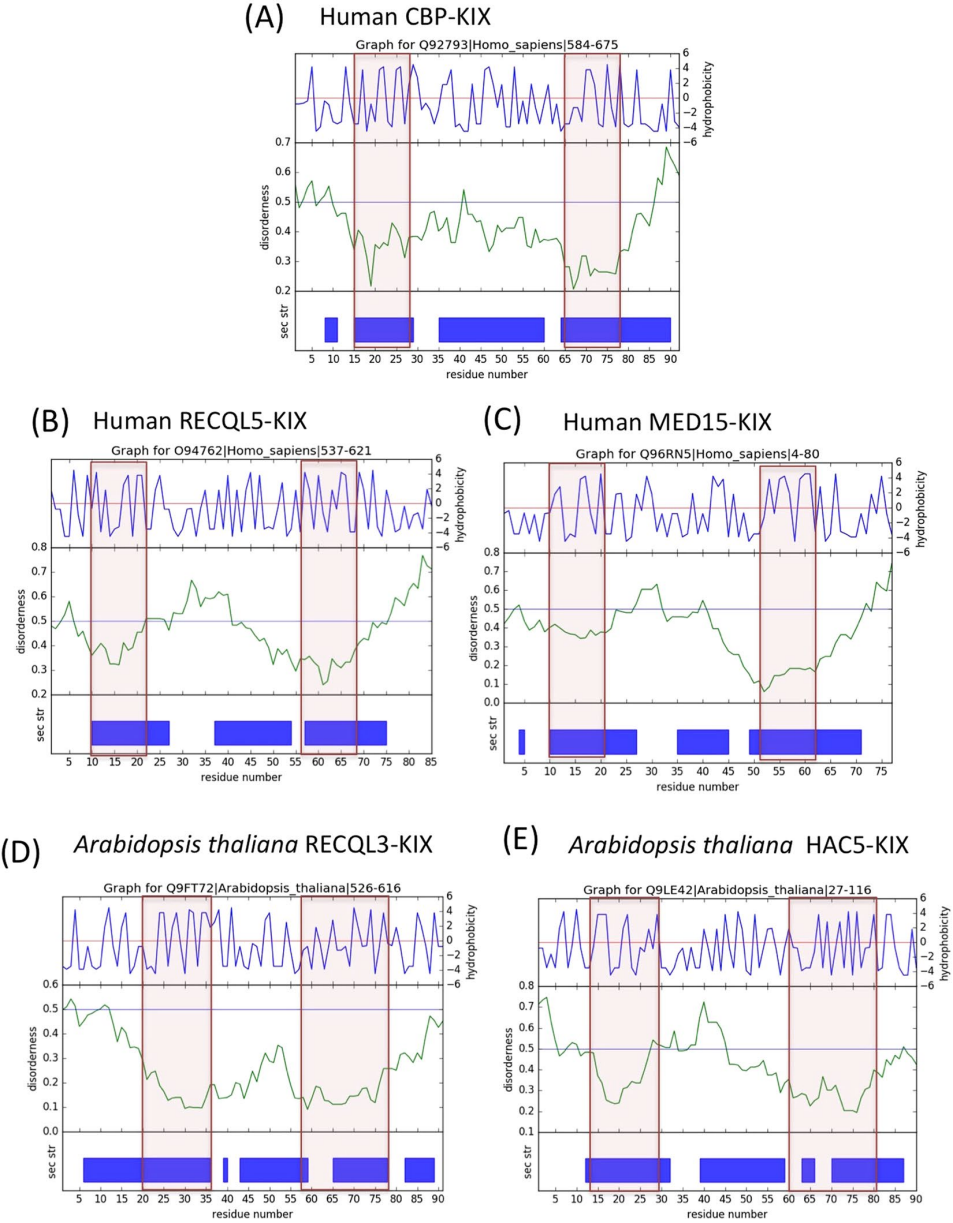
Comparison of structural features among characterised KIX. domains. The common structural patterns in multi feature plots of Human (**A**) CBP, (**B**) RECQL5, (**C**) MED15, (**D**) *Arabidopsis thaliana* RECQL3 and (**E**) *Arabidopsis thaliana* HAC5 can be observed here.

The binding site residues of KID and c-Myb sites were mapped to the multi-feature plots to better understand the relationship between KIX-KID/c-Myb site with its internal disorder and secondary structure. As represented in Fig. 5B,C, we found that the regions comprising KID/c-Myb binding site residues at alpha-1 and alpha-3 helices were associated with a noticeable dip in the internal disorder as compared to the other parts of KIX domain including alpha-2 helix. This observation indicates a higher thermodynamic stability of the binding site region in KIX domain owing to its functional relevance. A study based on the structural analysis in CBP-KIX reports that C-terminal of the alpha-3 helix, beginning from Tyr-657 is completely unfolded and gains helicity upon binding with TAD^30^. This can also be easily demonstrated in the multi-feature plot where the C-terminus of alpha-3 seems to be highly disordered compared to the rest of the KIX domain. A scan of several such plots via KIXBASE, allowed us to detect the pattern of disordered regions to be quite conserved in all CBP, Med15 and RECQL5 KIX domains in human and mouse, as shown in Fig. 6A–C. Examples of such a pattern can be seen in plants as well (Fig. 6D,E). This is further reiterated by a simple C-alpha based structural superimposition of the available 3-D structures of KIX domains from each class, revealing their RMSDs to be 1.77 (between CBP and MED15 KIX), 2.02 (between CBP and REC KIX) and 1.82 (between MED15 and REC KIX) over aligned residues, suggesting structural conservation. The PDB IDs used for this structural superimposition were 1SB0 (CBP), 2GUT (MED15) and 5LB3 (RECQL5). Interestingly, the huge dataset of KIX domains identified in metazoans, fungi and plants enabled us to assess the extent of sequence conservation between and within KIX domains from various forms of life and Table 4 depicts the average %identity thus found.

**Table 4:** Average sequence conservation (%identity) of KIX domains within and between taxonomic groups.

	Metazoan KIX	Plant KIX	Fungal KIX
Metazoan KIX	73.36		
Plant KIX	28.01	50.63	
Fungal KIX	26.39	28.12	41.82

This data provides evidence that KIX domains indeed have higher sequence similarity within their own class and that homology is difficult to detect between taxonomic kingdoms, as stated earlier in the introduction. In summary, this short case study shows that although KIX domains might not be sequentially well conserved among different species and classes, they do share common structural features, emphasising the importance of structure-based studies for KIX domain analysis, a feature that is unique to KIXBASE and lacking in other domain databases.

## Discussion

Regulation of transcription is one of the most crucial steps in the gene expression assisted by the interplay of proteins, which interact with each other and with DNA. KIX domain is directly involved in this process, acting as a docking site for transcription factors and nuclear receptors. Presently, researchers can only depend on general composite databases like Pfam and InterPro for KIX identification and retrieval, which are unable to provide enough analytical information about each KIX record. Furthermore, these databases do not sufficiently represent KIX domains for plants and other lower organisms. As emphasised before, the RECQL5 KIX records are missing altogether in these databases. Thus, there is a need for a database that is completely dedicated to the identification and detailed analysis of KIX domains in all kingdoms of life, and KIXBASE has been developed to fill this lacuna. KIXBASE is a novel database and a multi-analysis portal for KIX domains, which can be of great utility to researchers working in this field. Through KIXBASE, a vast amount of data related to KIX domains can be accessed from a single web resource, constantly updated by NIPGR. It may be noted that KIXBASE contains both BLAST as well as HMM based search protocol for input seqeunces. The BLAST method is for querying user input to the database that was constructed using the HMM search. Therefore, both portals would give similar results although BLAST would perform faster. The predictive web server would be useful for detecting new KIX-containing proteins that are not already in our curated database, resulting in consistent performance. In either case, KIXBASE not only helps in identification of KIX domains but also further annotates them through conservation and structural features. All KIX domains are aligned with characterised KIX domains present in PDB to get a viewpoint on its conservation. Secondary structure for all the KIX domains are represented and triple helix states can be visualised with each residue confidence scores. Users can perform further analyses like checking the state of internal disorder in each helical region, an aspect related to KIX binding through multi-feature plot analysis. KIXBASE also helps in annotating uncharacterised proteins containing KIX domains, especially in plants by providing information about domain architectures. This first version of KIXBASE does not classify predicted KIX domains into the three known categories of CBP, MED15 and RECQL5, due to the high amount of sequence and structure conservation between these classes, but we hope to add this feature in future versions of KIXBASE with increasing experimental data for each KIX class. In plants, sequence similarity between KIX domains sometimes highly varies among the species and quite often CBP, MED15 and REC KIX are confused due to lack of proper annotations. With KIXBASE, we studied the largest online repertoire of KIX domains for class-specific patterns and found that in general, CBP and CBP related KIX domains are accompanied with HAT, TAZ, bromodomain and/or ZZ domains whereas, MED15 and MED15 related KIX are accompanied by MED 15 and protein kinase/kinase-like domains. Similarly, RECQL5 and homologues contain Helicase C, RecQ5, RecQ_Zn_binding and/or DEAD domains. Interestingly, we have identified proteins with standalone KIX domains, or up to six tandem KIX domains in a protein sequence in *Brassica oleracea*. Such extensive information about KIX domains cannot be obtained anywhere else as of date and we also hope to use the presence or frequency of selected neighbouring domains as a marker to identify KIX category. All data and records in KIXBASE are arranged in a systematic user-friendly manner, which is easy to navigate and retrieve.

Various benchmarks and statistical performance tests were conducted in this work, and despite not being a perfect predictive algorithm, KIXBASE can be safely recommend as a method of choice for biologists interested in KIX domain anlaysis, as compared to available general annotation tools like Pfam, Interpro and Prosite. In view of the statistical measures depicted in Tables 1 and 2, we would like to add that it is indeed possible for KIXBASE to predict remote homologs which may qualify as false positives, when run on whole proteome, but the KIXBASE webserver supports each prediction with secondary structure, intrinsic-disorder, and alignments with characterized KIX domains, and these features can assist users to critically analyze the prediction. It must also be rememebred that the KIX domain has a tendency to occur in diversely non-homologous proteins and therefore there is vast scope of novel predictions for KIX in biological research. In future versions of KIXBASE, we hope to incorporate domain classification and comparative structural analysis on KIX domains present across all forms of life. KIXBASE can provide useful information for functional characterization through mutational studies. They can be used to identify KIX-TAD interacting residues and help understanding complex protein interaction networks during transcription.

## Material and Methods

### KIX domain identification

The KIX identification algorithm uses profile Hidden Markov Models. The initial dataset used for generation and training of profile HMMs was compiled by extraction of FASTA sequences of proteins containing KIX domain in various species via a keyword-based search (i.e. KIX, CBP KIX, KIX domain) of various protein family databases, such as Pfam, PROSITE, Superfamily, SMART and Gene3D, which are collectively present in InterPro Consortium. This dataset was filtered for redundancy using the CD-HIT^33^ program at 90 percent sequence similarity. Regions corresponding to KIX domain in the non-redundant dataset sequences were extracted using in-house perl scripts, to generate the training set. Characterised KIX domains of RecQL5, Yeast GAL11p, ARC105 and recently identified plant KIX domains (AT1G15780, AT1G15790, AT1G16705, AT1G16710, AT1G7900, AT2G10440, AT4G35740 and AT3G12980 of *Arabidopsis thaliana* and LOC_Os01g14370, LOC_Os02g04490 and LOC_Os04g03860 of *Oryza sativa*) were added to the training set to improve the accuracy of prediction^14^. In all, the training set contained 312 KIX domains, all of which have been provided to KIXBASE users on the website under Tutorials/Datasets section. The HMM profile for KIX domains was generated using HMMER v3.1b1^34^. This profile was run on the complete proteomes of 118 plants, 278175 metazoans and 924 fungal species, extracted using NCBI RefSeq^23^, UniProtKB^24^ and Phytozome-10^25^. As shown in the workflow in Fig. 1, the results were filtered for ‘significant’ predictions with a maximum E-value cut-off of 0.01 and minimum length cut-off of 70 residues. Further filtering to remove false positives was performed by fold recognition using MUSTER (I-TASSER)^26^ in only retain records with highest fold similarity to any of the characterised KIX domain available in the PDB^35^ library. This incorporation of fold recognition feature makes KIX database highly accurate and reliable.

### Benchmarking and web interface

Cross-validation is a mode of performance estimation for a predictive model on a dataset, which is not used for generating it. It involves partitioning the data into complementary sub-sets, developing the predictor on one sub-set (the training dataset), and validating the prediction efficacy on the other sub-set (called testing dataset). To reduce variability due to sample partition, multiple rounds of cross-validations are performed using different data partitions and results are averaged over all partitions. Three of the most common cross-validation methods used in statistical prediction are independent dataset test, sub-sampling test and jackknife test. Of these, the Jacknife test (also called the ‘leave-one-out’ test), has been increasingly and widely used by investigators to examine the quality of various predictors and has also been shown to be a method of choice for evaluation of hidden markov models, since it is considered least arbitrary and can yield a unique result for a given benchmark dataset whereas the other two test methods bear considerable arbitrariness, as elaborated by Chou in a detailed analysis that we strongly recommend to interested readers^31^.

For evaluating the KIXBASE pipeline, a negative dataset was prepared by collecting TFIIS sequences and its homologs through BLAST. TFIIS was selected on account of its marked structural similarity with RecQL5 KIX. As mentioned previously, RecQL5 KIX mimics TFIIS structure and binds to RNA pol II instead, thus blocking transcription elongation. In addition, we also took KIX structural homologs through DALI server^36^ since sequences closely related to KIX would serve as a suitable negative dataset. The final negative test set comprised of 1144 sequences. The training set was based on known KIX sequences which were used to build a multiple sequence alignment (MSA) that was used as input to the HMMER package through *hmmbuild* option.

#### Cross-Validation Test

The jackknife cross-validation experiment was conducted on KIXBASE to validate the HMM performance for detection of sequences not used in the creation of the profiles (pseudo-novel sequences). In order to follow the jackknife model testing procedure, we consider as “full data set” all members of the HMM seed set plus the 1144 negative examples. In each iteration, one sequence was removed from the MSA of the HMM’s seed set, and a new HMM was constructed from the remaining sequences in the MSA. Then the profile’s ability to correctly classify the removed sequence was measured. To test the negative dataset, this involved x + 1144 iterations, where x is the size of our HMM seed set. For 1144 of these iterations the HMM was identical (since no members of the positive set are being held out), but the point is still to have symmetric treatment of the positive and negative examples in the designated training set, and to test the classification of exactly one data point (the one that is held out) at each iteration. This was followed by assessment of the following statistical parameters for evaluation and performance:

$${\rm{Sensitivity}}={\rm{TP}}/({\rm{TP}}+{\rm{FN}})$$

$${\rm{Specificity}}={\rm{TN}}/({\rm{TN}}+{\rm{FP}})$$

$${\rm{Precision}}={\rm{TP}}/({\rm{TP}}+{\rm{FP}})$$

$${\rm{Accuracy}}=({\rm{TP}}+{\rm{TN}})/({\rm{TP}}+{\rm{FN}}+{\rm{TN}}+{\rm{FP}})$$

$${\rm{MCC}}=(({\rm{TP}}+{\rm{TN}})-({\rm{FP}}+{\rm{FN}}))/\sqrt{(({\rm{TP}}+{\rm{FN}})\cdot ({\rm{TP}}+{\rm{FP}})\cdot ({\rm{TN}}+{\rm{FN}})\cdot ({\rm{TN}}+{\rm{FP}}))}$$

$$({\rm{MCC}}={\rm{Matthew}}\mbox{'}{\rm{s}}\,{\rm{Correlation}}\,{\rm{Coefficient}}).$$

In general, sensitivity is the proportion of positive sequences correctly identified as KIX whereas specificity is the proportion of negative sequences correctly identified as non-KIX. Precision is a fraction of retrieved positives that are relevant and accuracy is an overall population of true results in the population. MCC is considered the best measure of the quality of classification when the test datasets are of different sizes during binary classification. MCC ranges from −1 to 1, where values closer to −1, 0 and +1 depicts poor, random and good predictions respectively.

In order to support our claim that KIXBASE is superior to global general databases for KIX domain assignment, we have performed Jackknife tests for the two Pfam profiles related to KIX, namely, pfam KIX and pfam KIX-2 where the same negative test set of 1144 sequences was used as in case of KIXBASE above, along with each sequence in the respective MSA as iterative positive test data. Hence our positive set (x) for generating Table 1 included 388 sequences (312 for KIXBASE, plus 5 and 71 respectively for the two Pfam profiles). In addition, performance comparison between KIXBASE and Global protein databases was also carried out using independent datasets for Humans, Yeast and Arabidopsis for generating Table 2. The positive test set contains known Med15, CBP and RECQL proteins containing complete/partial KIX domain from uniprotKB. The Negative test set contained all reviewed (swissprot) protein sequences with molecular function GO term ‘not’ related to transcription from uniprotKB.

### Development of KIX Web Server and Database

For the development of the web repository, all KIX domain regions were mapped onto FASTA sequences and highlighted using python code. PSIPRED 3.5^27^ was used for prediction of secondary structures of KIX sequences. The UniRef90^37^ dataset was downloaded in order to run psiBLAST^38^ search required for PSIPRED runs. A python script was developed to generate 2D plots from result scores of PSIPRED, IUPRED^39^ and hydrophobicity Kyte-Doolittle index^40^ for KIX domains. The python script included Numpy^41^, pylab and Matplotlib^42^ packages for plotting and graphics environment. Each KIX domain sequence was aligned with a parent set of characterised KIX domains using the Clustal Omega^28^ program. Domain arrangements plots were generated through a highly efficient pipeline where each sequence was scanned for known domains through ‘hmmscan’ program from HMMER followed by E-value filters and overlap removals. Pfam-A seed alignments were utilized for searching additional annotated domains present in combination with predicted KIX domains on a given sequence. PHP, CSS and HTML programming languages were employed to develop the KIXBASE user interface, with output in the form of tables and coloured plots generated through manual scripts. BLASTP^43^ service by NCBI is also utilised to search a query across the database. In house perl scripts were developed to color the alignments in accordance with conventional Clustal-O coloring scheme based on physiochemical properties. MSA package from Bioconductor was used to generate conservation-based display of alignments^44^. A total of 326 metazoans, 170 fungi and 90 plant species are present in the KIXBASE. The complete list of these species along with classification tree can be found in KIXBASE. The training and test sets used for generating and testing profile HMM are also available for download on the website under the Tutorial/Dataset link. The web portal is freely available to the scientific community, at http://www.nipgr.res.in/kixbase/home.php.
